# *Serratia*‐based toxin cluster elements are associated with a type I fimbria

**DOI:** 10.1002/mbo3.1395

**Published:** 2024-01-02

**Authors:** Lesley Sitter, Marion Schoof, Travis R. Glare, Murray P. Cox, Peter C. Fineran, Paul P. Gardner, Mark R. H. Hurst

**Affiliations:** ^1^ Resilient Agriculture, AgResearch, Lincoln Research Centre Christchurch New Zealand; ^2^ Bio‐Protection Research Centre Lincoln University Lincoln New Zealand; ^3^ School of Natural Sciences Massey University Palmerston North New Zealand; ^4^ Department of Statistics University of Auckland Auckland New Zealand; ^5^ Bioprotection Aotearoa Lincoln University Lincoln New Zealand; ^6^ Genetics Otago Research Centre University of Otago Dunedin New Zealand; ^7^ Department of Microbiology and Immunology University of Otago Dunedin New Zealand; ^8^ Department of Biochemistry University of Otago Dunedin New Zealand

**Keywords:** fimbria, grass grub, plasmid, repA, Serratia, toxin complex

## Abstract

A soil bacterium in the *Serratia* genus, carrying a 153 kb conjugative amber disease‐associated plasmid (pADAP), is commercially exploited for population control of the New Zealand endemic pest beetle *Costelytra giveni* (Coleoptera: Scarabaeidae). The main insecticidal elements are an anti‐feeding prophage and the Sep ABC toxin complex (Tc). Homologs of pADAP, encoding variant Tcs, convey different beetle disease phenotypes. To investigate the correlation between variable bioactivity and the Tc variant, 76 *Serratia* plasmids were sequenced, resulting in the identification of four additional *tc* variants. All *Serratia tc* variants were found to be colocated with a conserved type 1 *sef* fimbrial‐like operon, indicating a conserved *sef‐tc* genetic island not observed outside of the *Serratia* genus. The conserved co‐location of the fimbrial and *tc* genes suggests the fimbriae somehow contribute to the lifestyle of Tc‐producing cells. Expression of the *sef* operon in a fim‐null *Escherichia coli* strain revealed fimbriae presence while a constructed *sef*‐deficient mutant showed no reduction of virulence or host colonization. Although no detectable contribution of Sef to amber disease in *C. giveni* was observed, the Sef adhesin sequences clustered similarly to the *Serratia* species encoding it, suggesting Sef has a species‐specific function.

## Graphical abstract

This article characterizes Sef fimbrial elements that have been observed to be consistently colocated with an ABC toxin complex (Tc) found in pathogenic Serratia species that infect the beetle larvae of *Costelytra giveni*. This unique association between the fimbria and Tc has not been documented before in any known Tc system and raises questions about the high selective pressure for maintaining these fimbriae only in bacteria that encode Tcs. Our study also sought to characterize the structure and function of Sef fimbria using bioinformatics, cloning, and experimental approaches.

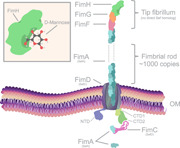

## 1 INTRODUCTION

To prevent substantial damage to crops, synthetic insecticides are often utilized to treat fields from insect infestations. Recent studies have shown that these synthetic pesticides can have detrimental side effects including contaminating groundwater (Akay Demir et al., 2019) and rivers (Iwafune, 2018); lethal nontargeted effects on other organisms such as birds (Kwon et al., 2004) and fish (Muñoz‐Arnanz et al., 2019); harm to beneficial insects (Pisa et al., 2015); reducing the fertility of the soil (Zaller et al., 2016); negative health effects on farmers (Mrema et al., 2017; Sak et al., 2018) and consumers (Ma et al., 2019); and other effects (Aktar et al., 2009). This has led to the increased use of biological control products such as commercially exploited strains of *Bacillus thuringiensis* which produce distinct Cry toxins with bioactivity in various invertebrate hosts (Palma et al., 2014). In New Zealand an insecticidal strain of *Serratia entomophila* has been developed into biopesticides (Jackson & Glare, 1992; Johnson et al., 2001) for the control of larvae of the New Zealand endemic pest beetle species *Costelytra giveni* (Coleoptera: Scarabaeidae) (Coca‐Abia & Romero‐Samper, 2016; Given, 1952). The larvae of *C. giveni*, also known as tūtae ruru in Māori and more commonly referred to as grass grub, are a widespread pasture pest, impacting New Zealand's primary sector (Ferguson et al., 2019). The *S. entomophila* A1MO2 amber disease‐associated plasmid (pADAP) (Glare et al., 1993) encodes two known virulence determinants (Hurst et al., 2011) that have been shown to induce amber disease in *C. giveni* larvae (Grimont et al., 1988). The two virulence determinants causal to amber disease are the Anti‐feeding prophage (Afp) contractile injection system (Hurst et al., 2004), and the *S. entomophila* pathogenicity (Sep) ABC toxin complex (Tc) (Hurst et al., 2000). The *Serratia proteamaculans* AGR96X plasmid, pAGR96X, encodes an Afp variant termed AfpX which has high and rapid activity against *C. giveni* larvae as well as two other Scarabaeidae, *Pyronata festiva*, and *P. setosa* (Hurst et al., 2018) but does not encode a *tc* cluster (Hurst et al., 2018; Sitter et al., 2021).

The *S. entomophila* pADAP Sep Tc is a member of the ABC Tc family first identified in *Photorhabdus luminescens* (Bowen et al., 1998; Hurst et al., 2000). ABC Tcs consist of three components, TcA, TcB, and TcC, which have since been identified in a wide range of microbial species including *Xenorhabdus nematophilus* (Morgan et al., 2001), *Yersinia frederiksenii* (Dodd et al., 2006), *B. thuringiensis* (Blackburn et al., 2011) and *Pseudomonas fluorescens* (Rangel et al., 2016). In these ABC complexes, the TcC component is the toxic effector (Hurst et al., 2021; Zhang et al., 2012). The C‐terminus of the TcC effector is enveloped by TcB and docks to the TcA pentamer (Busby et al., 2013). The encapsulation of TcC is assumed to protect the producer cell from the cytotoxic effects of the TcC component before its release (Busby et al., 2013).

The *S. entomophila* A1MO2 type strain that encodes both Afp and Sep Tc on pADAP causes 95%–100% amber disease in challenged *Costelytra giveni* larvae (Glare et al., 1993), while the *Serratia proteamaculans* pathogenicity (Spp) Tc variant encoded on the *S. proteamaculans* plasmid pU143, which is devoid of Afp, affects ~70% of the challenged larvae (Hurst et al., 2011). The *Y. frederiksenii* 49 49.p1 encodes the ABC toxin complex *Y. frederiksenii* (TcYF) operon, which shares 79.5% and 73.4% nucleotide sequence similarity to the pADAP *sep tc* and pU143 *spp tc* operon respectively, yet *Y. frederiksenii* 49 is ineffective towards challenged *C. giveni* larvae (Dodd et al., 2006). Other *Serratia* Tc encoding plasmids include the *S. proteamaculans* isolate p159 (Dodd et al., 2006), Man 4 and C (Hurst et al., 2021) all presenting varying bioactivity towards *C. giveni* and *Pyronota* species. The observed variation in host ranges and virulence phenotypes of *Serratia* isolates led to 76 *Serratia* isolates being sequenced and screened for the presence of additional pathogenicity‐associated genetic components (Sitter et al., 2021). In silico analysis of these 76 *Serratia* isolates showed that 68 isolates carried one or more plasmids, with a total of 96 plasmids identified. Fifty of the 96 plasmids contain a ~62 kb conserved backbone, homologous to that of pADAP, within which divergent accessory determinants were located. Accordingly, these 50 plasmids were designated as *Serratia* transmissible adaptive mega‐plasmids (STAMP) (Sitter et al., 2021). Twelve STAMP variants, labeled A–L, were identified, each encoding different accessory regions. The study of STAMPs identified a further four distinct *tc* clusters in STAMP type H, I, J, and K, each with differing activity toward *C. giveni, P. festiva*, and *P. setosa* (Sitter et al., 2021), increasing the number of known *Serratia* Tc types to 11. These STAMP plasmids are characterized by a conserved *repA* replication gene, with its closest orthologues coming from *Edwardsiella tarda* and *Salmonella enterica*. The STAMP backbone comprises a replication and partitioning region followed by a relaxosome operon, with no direct homolog (≥50% nucleotide identity) outside of the *Serratia* system. The STAMP backbone also encodes a type IV pili encoding *pil* operon which is, in part, homologous to regions found on *Salmonella* Typhimurium plasmid R64, *Salmonella* Infantis strain 119944 plasmid pESI and *Yersinia ruckeri* strain NHV_3758 plasmid pYR4 (Sitter et al., 2021).

The *S. entomophila* A1MO2 Sep *tc* operon is encoded 3′ of a type 1 fimbrial cluster, designated as the *S. entomophila* fimbria (Sef) (Hurst et al., 2011), and 5′ of the plasmid backbone. Members of the type 1 fimbriae, such as the *Escherichia coli* fimbriae FimA–H (Busch et al., 2015; Spaulding et al., 2018, Figure A1), are commonly associated with microbial colonization of mammals such as humans (Knight & Bouckaert, 2009; Müller et al., 2009), mice (Connell et al., 1996) and swine (Althouse et al., 2003), and even invertebrates including nematodesand insects (Chandra et al., 2008).

This study reveals a strong co‐location between Tcs and the Sef fimbria encoding cluster in both documented STAMPs and newly characterized Tc‐encoding non‐STAMP *Serratia* plasmids. Interestingly, *Serratia* Tc‐encoding operons are always found to be colocated with a *sef* cluster, while no *sef* clusters were identified with other pathogenic islands such as those encoding Afp or AfpX, or other STAMP accessory determinants like RUF operons. In this study, using targeted mutagenesis, gene expression, transmission electron microscopy (TEM), and comparative genomics, we examined whether or not Sef aids pathogenicity, and aimed to identify the function of the fimbrial cluster to further define the evolution and role of *tc* and *sef* operons in the *Serratia* system.

## 2 RESULTS AND DISCUSSION

### 2.1 Identification of genes encoding toxin complexes on non‐STAMPs

In an attempt to identify novel *Serratia‐based* Tcs and signatures of horizontal gene transfer of Tcs, we analyzed 36 plasmids that did not share any similarity with the STAMP backbone (Sitter et al., 2021). Five additional *tc* clusters were identified on the plasmids *S. proteamaculans* S‐prot‐1 S‐prot‐1.p1, D1 D1.p1, Man4 Man4.p1, M3 M3.p1 and Sm1a Sm1a.p2 (Supporting Information S1: Table S1). These five new non‐STAMP Tc‐encoding plasmids all encode an incFII family plasmid replication initiator *repA* gene homologous to several *repA* genes found on plasmids in *Yersinia* species, yet sharing little nucleotide similarity to the *repA* on pADAP (Figure A2).

Although the *Y. enterocolitica* LC20 plasmid1_80K encodes the most distant of all these *repA* nucleotide sequences (Figure A2), its predicted ~15 kb backbone shares >72% nucleotide identity with the predicted plasmid backbones of the non‐STAMP plasmids Man4.p1, S‐prot‐1.p1, D1.p1, M3.p1, and the previously published 591.p1 (Figure A3). The backbone region of these five non‐STAMPS Tc‐encoding plasmids comprises an array of 20 *tra* genes and three *trb* genes, orthologs of which are implicated in transmissibility of plasmids (Kurenbach et al., 2002), as well as several hypothetical proteins and mobile genetic elements (Figure A3). The Tra/Trb encoding region of these non‐STAMPs is different from that found on the STAMP backbone (Sitter et al., 2021) and no STAMP‐like type IV pili or partition stability loci were identified on the backbones of these five Tc encoding non‐STAMP plasmids, further differentiating these plasmids from STAMPs. The Sm1a.p2 plasmid encoded a different backbone.

The nucleotide similarity between the backbone of the five non‐STAMP Tc encoding plasmids and the *Y. enterocolitica* LC20 plasmid1_80K, coupled with the low nucleotide identity of the *repA* gene to that of pADAP, suggest that the acquisition of Tc by these non‐STAMPs may have occurred through a shared ancestral plasmid common to the Yersiniaceae family. To understand how these non‐STAMPs acquired their *tc* operons, we analyzed the flanking regions of these operons.

### 2.2 Ancestral connection between Sef and Tcs

**Figure 1:**
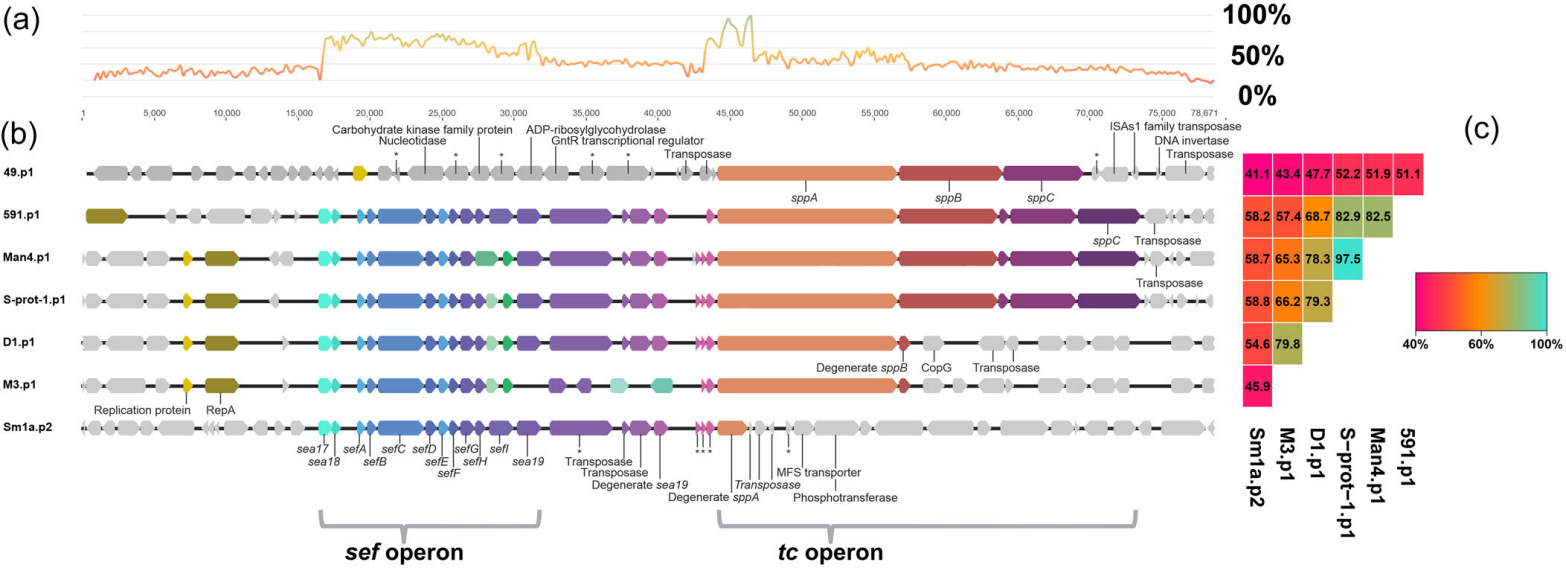
Maps of the *sef* and *tc* encoding area on the non‐*Serratia* transmissible adaptive mega‐plasmids (STAMP) plasmids. (a) The nucleotide similarity chart of the six non‐STAMP plasmid encoding Tc elements. (b) These are the feature maps of the largest plasmids of *Serratia proteamaculans* pD1, pS‐prot‐1, p591, pMan4, *Serratia entomophila* pM3 and *Yersinia frederiksenii* 49.p1, and the second largest of *S. proteamaculans* pSm1a. Homologous genes are denoted by the same color. (c) A heatmap showing the nucleotide identity (%) between the Sef and Tc encoding regions of all depicted plasmids.

To identify the demarcation sites of the *tc* islands in the six non‐STAMPs (*S. proteamaculans* D1.p1, S‐prot‐1.p1, Sm1a.p2, Man4.p1, 591.p1 and *S. entomophila* M3.p1), we looked at the surrounding regions of the Tcs. Surprisingly, unlike the avirulent *Y. frederiksenii* 49.p1, all non‐STAMP Tc encoding *Serratia* plasmids encode a conserved *sef* operon 5′ of the Tc encoding cluster (Figure 1, Supporting Information S1: Table S2). Although the gene arrangement of non‐STAMP encoded Sef gene clusters is highly conserved, their corresponding *tc* clusters exhibit lower conservation, as exemplified by *S. proteamaculans* Sm1a.p2, which only encodes the first 1848 bp of *tcA* instead of the full 7221 bp of its closest homolog *S. proteamaculans* pU143 TcA, and no TcB of TcC elements.

The co‐location of the *sef* and *tc* operons has previously been noted in pADAP (Hurst et al., 2011) and more recently in several groups of the greater STAMP family (Sitter et al., 2021). During the previous analysis of STAMPs, it was thought that the presence of most *sef* and *tc* regions in STAMPs was a result of differentiation following two separate acquisitions by the STAMP backbone (Sitter et al., 2021). The co‐location of *tc* and *sef* in both non‐STAMP and STAMPs suggests that the *tc* and *sef* operon likely comprise a single genetic island that has been horizontally transferred between different *Serratia* plasmids, further corroborated by the presence of transposons and IS elements flanking the *sef‐tc* region, indicative of a hotspot for mobile genetic material.

**Figure 2:**
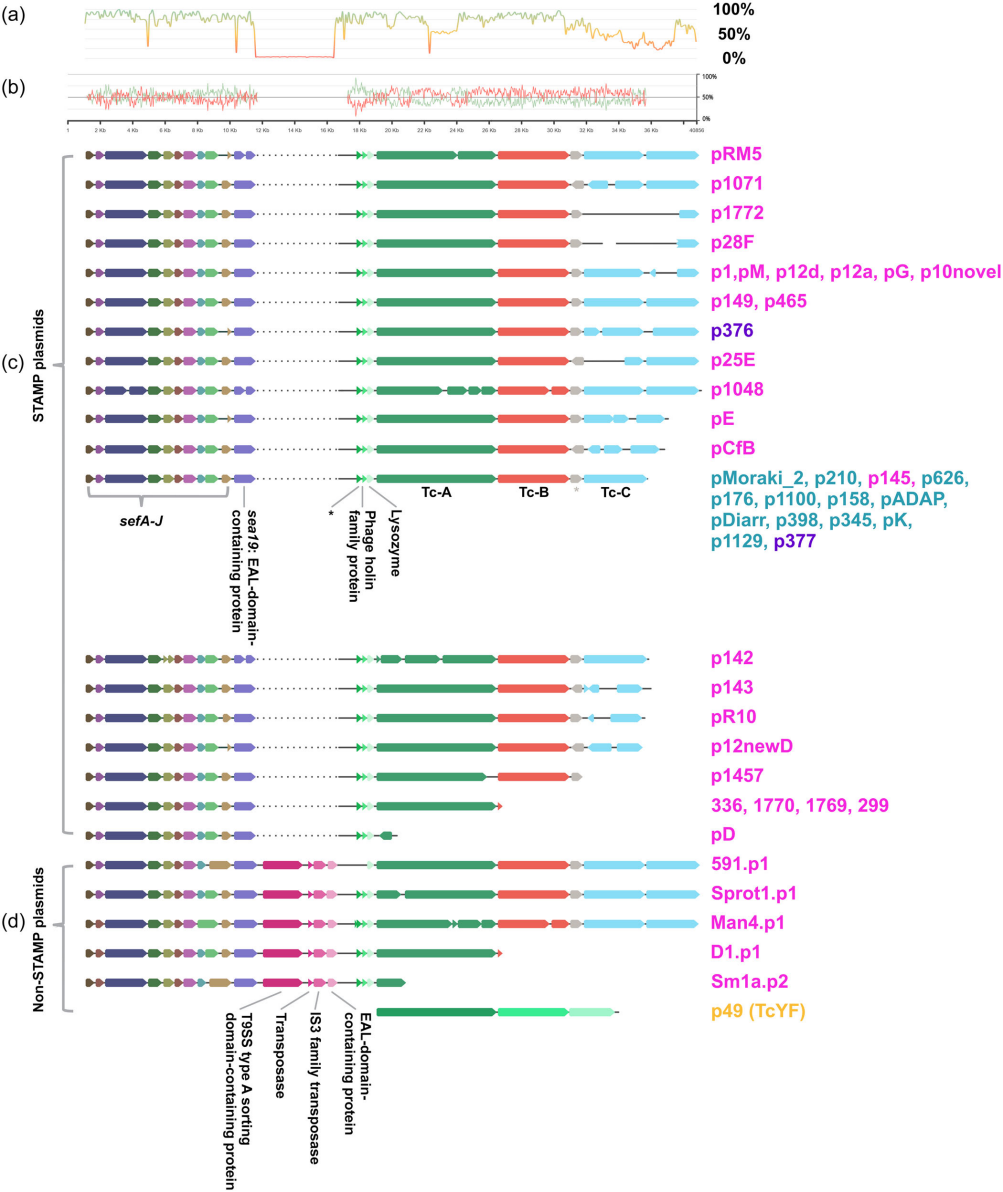
DNA alignment of all *sef* ‐*tc* orthologous regions. (a) Nucleotide chart showing nucleotide sequence identity for the alignment of all sequences. (b) GC plot for the pADAP‐based Sef‐Tc encoding region, where GC is red and AT is green. (c) *Serratia* transmissible adaptive mega‐plasmids (STAMP) based *sef*‐*tc* clusters. Dotted lines indicate gaps in the alignment. (d) non‐STAMP‐based *sef*‐*tc* clusters. Plasmid names are shown on the right and are colored according to their respective host species as follows, turquoise, *Serratia entomophila*; purple, *Serratia liquefaciens*; pink, *Serratia proteamaculans*; yellow, *Yersinia frederiksenii* (does not encode fimbria on its plasmid). Additional isolate information is found in Supporting Information S1: Table S1.

The Sef‐Tc encoding island nucleotide alignment revealed that *tcB* is the most conserved gene in the *tc* operon (average 97.5% nucleotide identity) compared to the *tcA* (average 75.7% nucleotide identity) and *tcC* (average 63.4% nucleotide identity) (Figure 2). Direct nucleotide sequence comparison of the *tcC* components is hindered by several Tcs encoding for two TcC components and some, such as the *S. proteamaculans* D1.p1, Sm1a.p2, p1457, pD, p336, p1770, p1769, and p299, lack the *tcC* altogether. Additionally, the 3′ region of the *tcC* genes is highly divergent (Hurst et al., 2021). Higher variation in the *tc* clusters may be a result of selective host or environmental pressure. The high rate of genetic drift of the *tc* operons could mean some of these bacteria no longer have pathogenic interactions with *C. giveni* larvae.

The in silico analysis of the STAMP and non‐STAMP plasmids' *sef*‐*tc* islands boundaries revealed the last conserved elements to be the *sea17* and *sea18* genes 5′ of the *sef* operon. Additionally, a degenerate IS element was identified by Hurst et al. (2011), with the sequence 5″ GACTGCCCCACGTTTTTTCATGCTCTCCTA 3″, approximately 240 bp 5′ of *sea17*. Together *sea17*–*18* and the degenerate IS element demarcate the 5′ side of the putative *sef*‐*tc* island. As there were no functionally described homologous sequences identified to *sea17* and *sea18* in the NCBI Reference Sequence database (RefSeq), Phyre2 (Kelley et al., 2015) and InterProScan (Jones et al., 2014) were used to try and identify homologous protein domains. The presence of predicted regulatory motifs in Sea17 and Sea18 suggests that these proteins may be involved in the regulation of Sef (Supporting Information S1: Table S3). Assessment of the region 3′ of the *tc* operon of STAMP and non‐STAMP *tcs* revealed no shared mobile elements.

Except for the *Y. frederiksenii* 49.p1, each non‐STAMP encodes four additional genes 3′ of the *sef* operon and 5′ of the *tc* operon (Figure 2). These genes encode for a protein with a predicted type 9 secretion system domain, transposon, insertion sequence, and a 576 bp gene that shares >99% nucleotide identity to the 3′ portion of the EAL‐domain encoding *sea19* gene (652–1227 bp), and therefore appears to be a truncated paralog of *sea19*. Members of EAL‐domain‐bearing proteins have been implicated in biofilm regulation (Ahmad et al., 2013). The degenerate *sea19* indicates that these four genes have most likely originated from an insertion event at nucleotide position 652 of *sea19* as a result of homologous recombination, and therefore suggests that the *sef*‐*tc* island of these five non‐STAMPs evolved from one single common ancestor.

The presence of degenerate *tc* operons in some plasmids and a lower conservation of *tc* operons overall, compared to the high conservation of *sef*, indicates a higher selective pressure on the Sef elements. It is likely the acquisition of Sef only occurred once since the placement of *sef* 5′ of the *tc* operon is nearly identical for all plasmids. These findings could allude to the origins of the *sef*‐*tc* island most likely formed outside any of the currently known plasmid models before acquisition by either the backbones of STAMPs or non‐STAMPs.

**Figure 3:**
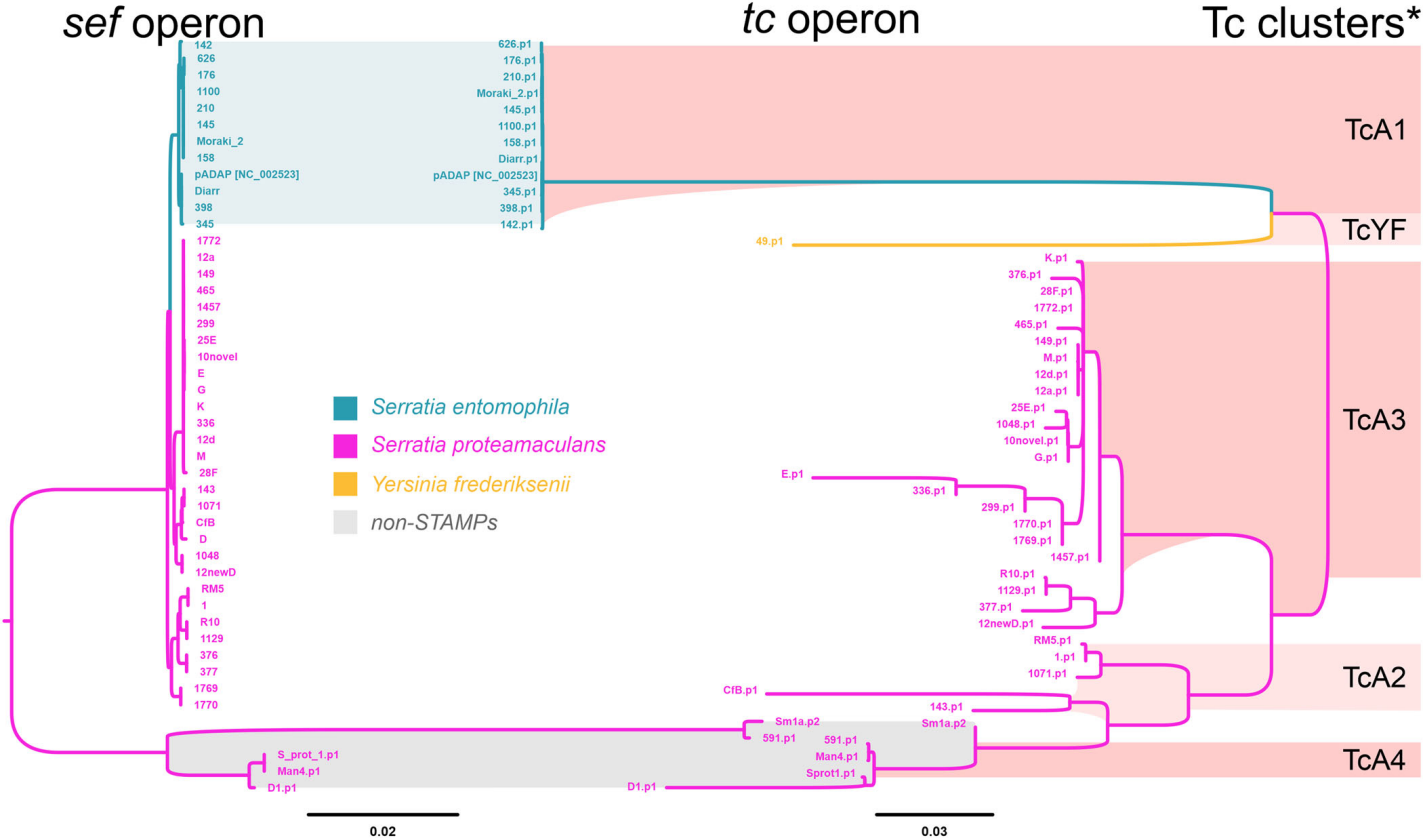
Phylogenetic clustering of the *sef* operon (left) and the corresponding *tc* operon (middle). Gray shade denotes non‐*Serratia* transmissible adaptive mega‐plasmids (STAMPs); blue *Serratia entomophila*; pink *Serratia proteamaculans*; yellow *Yersinia frederiksenii*. The right column denotes the different TcA clusters (Hurst et al., 2021).

An interesting finding was that phylogenetic analysis revealed both the *sef* and *tc* operons independently exhibit similar species‐specific clustering (Figure 3). This clustering further corroborates that the *sef* and *tc* operons evolved as one single island in a species‐specific manner instead of independently.

The findings presented so far raise the question of why the *sef* cluster is associated with different *tc* variants and why *sef* is not found colocated with other pathogenic elements such as the hypervirulent AfpX or AfpS encoding regions (Hurst et al., 2018) or the distinct RUF or N‐fixation cluster found on other *Serratia* plasmids (Sitter et al., 2021). To help elucidate the function of the *sef* operon, we analyzed the *sef* operon in more detail.

### 2.3 Characterization of Sef

**Figure A1:**
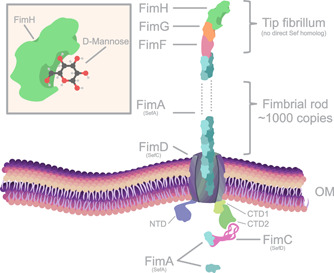
A simplified model of the type 1 fimbria typically found in *Escherichia coli*. FimC transports the FimH followed by FimG, FimF, and FimA proteins from the periplasm to a FimD usher in the OM. The FimD is an usher protein that forms a complex that helps transport the fimbria components through the OM into the EM. FimA is the protein that forms the fimbrial rod. The FimH tip has high binding affinity for D‐mannose, a residue found in glycoproteins on OMs. The Sef fimbria consists of mostly type 1 fimbria orthologs, although several genes, most notably the *sefH*, *sefII*, and *sefJ* appear to be relatively unique. The *sefH*‐*J* ORFs most likely form the tip fibrillum, which adheres to something other than D‐mannose residues. The figure is based on previously described models (Alonso‐Caballero et al., 2018; Busch et al., 2015; Godaly & Svanborg, 2007; Knight & Bouckaert, 2009; Kolenda et al., 2019).

**Figure A2:**
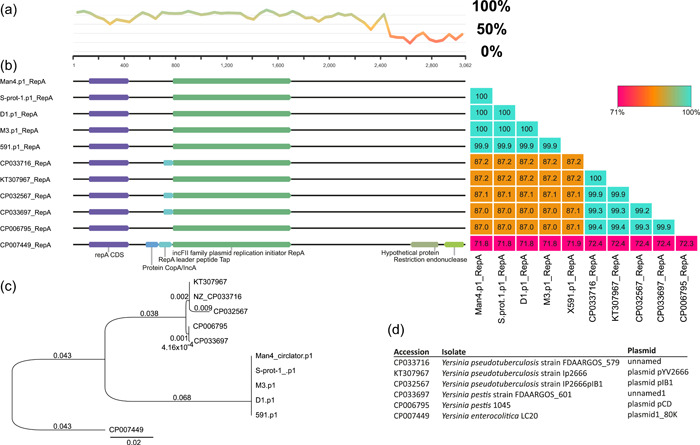
*repA* nucleotide alignment and phylogeny of non‐*Serratia* transmissible adaptive mega‐plasmids (STAMPs) to their closest RefSeq homologs reveal close similarity to *Yersinia*‐based *repA*. (a) The nucleotide similarity chart of the RepA‐encoding regions of Man4.p1, S‐prot‐1.p1, D1.p1, M3.p1, and Sm1a.p2 and their closest homologs using their respective RefSeq accessions as names. (b) The feature maps of the RepA encoding region of each plasmid with a heatmap depicting the relative percentage nucleotide identity among all sequences. (c) A maximum‐likelihood tree showing the relationship between all RepA encoding regions. (d) Reference table correlating the Accession IDs to the respective RepA encoding isolate and plasmid names.

**Figure A3:**
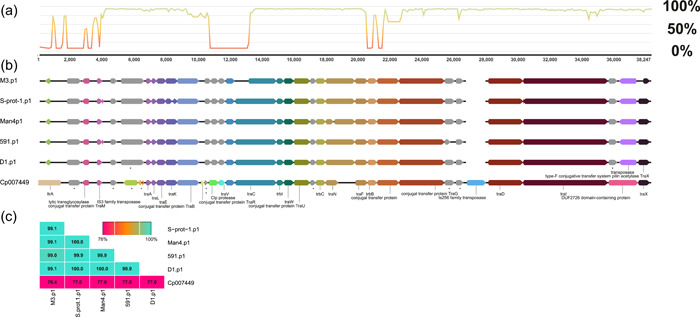
The ~15 kb Tra encoding backbone region of the Tc‐encoding non‐*Serratia* transmissible adaptive mega‐plasmids (STAMPs) and their closest orthologues identified in the study. (a) Nucleotide similarity chart of the backbone region of Man4.p1, S‐prot‐1.p1, D1.p1, M3.p1 and 591.p1 and their only homolog, *Yersinia enterocolitica* LC20 plasmid1_80K (CP007449). (b) Feature maps of the backbones of each plasmid with predicted gene function listed at the base. (c) Heatmap showing the percentage nucleotide identity between backbone regions of all depicted plasmids.

Based on gene identity, the *sef* operon is predicted to encode type 1 fimbriae, the best described of which is the *E. coli* type 1 fimbria (Figure A1), encoded by *fimA*–*H* (Busch et al., 2015), and Mrf fimbria found in several species of *Photorhabdus* (Meslet‐Cladiere et al., 2004).

**Figure 4:**
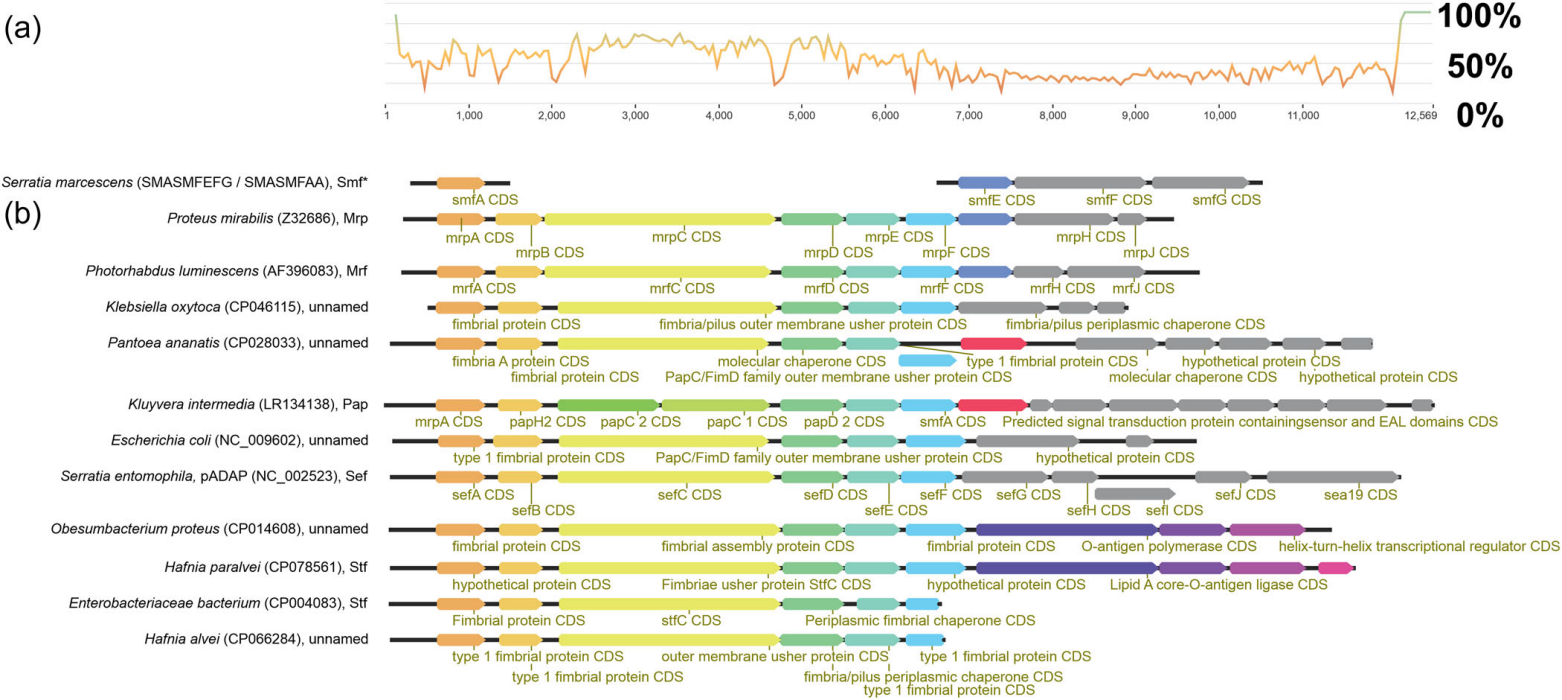
Gene synteny comparison of distant homologs of *sef*. (a) Nucleotide similarity chart where, although sharing similar gene synteny, there is low nucleotide conservation among similar fimbrial clusters depicted in (b). (b) Depicts the most homologous fimbrial clusters to the *sef* operon, as found in the NCBI RefSeq database. *Region was only partially sequenced. Sequences are ordered by pairwise nucleotide identity.

A search for *sef* operon orthologs reveals that *sef* is most closely related to the mannose‐resistant Mrf fimbriae (Figures 4 and A6) found in several species of *Photorhabdus* which have been implicated in facilitating infections of *Galleria mellonella* (Meslet‐Cladiere et al., 2004). The usher protein‐encoding genes homologous to *sefC*, are the most conserved among all closely related fimbrial operons.

SefD, SefC, and SefA are most likely a chaperone, usher protein, and fimbrial rod protein respectively based on homology to elements in other systems (Figure A1, Supporting Information S1: Table S4). The SefH amino acid sequence is orthologous to the *S. enterica* fimbrial minor subunit StfF of the Stf fimbria (de Masi et al., 2017) (Table 1). Though poorly characterized, Stf operon is upregulated within the chicken lumen (Harvey et al., 2011), implicating it in gut colonization, however, no significant difference in colonization capacity of a StfA knockout in the chicken intestine relative to wildtype was noted (Clayton et al., 2008). Both SefI and SefJ share low amino acid sequence similarity with adhesins of *Morganella* and *Proteus* (Table 1). Both *Morganella* and *Proteus* bacteria are often associated with mammalian microbiomes and, on rare occasions, have been implicated in mammalian diseases (O'Hara et al., 2000). As the role of these orthologous fimbrial proteins and adhesins has not been determined in these bacteria, combined with their limited sequence similarity to SefI and SefJ, we assume that SefI and SefJ have a role in adhesion to a yet‐to‐be‐determined substrate.

**Table 1:** Closest unique functionally described orthologs for SefH‐SefJ obtained using BlastX.

Protein	Hit						Accession	
SefH	MULTISPECIES: fimbrial protein (*Providencia*)						WP_109913237.1	
174 AA residues	Length:	170	Range:	25–170	Eval:	6E‐55		
	Ident:	85/146 (58%)	Pos:	106/146 (72%)	Gap:	0/146(0%)	Coverage:	83.0%
	Fimbrial minor subunit StfF (*Salmonella enterica* subsp. Salamae)						ECG8606686.1	
	Length:	158	Range:	7–158	Eval:	1E‐33		
	Ident:	71/159 (45%)	Pos:	99/159 (62%)	Gap:	9/159 (5%)	Coverage:	90.0%
SefI	Adhesin (*Morganella morganii*)						WP_107679199.1	
270 AA residues	Length:	272	Range:	158–272	Eval:	4E‐09		
	Ident:	44/117 (38%)	Pos:	61/117 (52%)	Gap:	6/117 (5%)	Coverage:	41.0%
	Adhesin (*Proteus columbae*)						WP_100158730.1	
	Length:	275	Range:	162–275	Eval:	6.00E‐09		
	Ident:	46/117 (39%)	Pos:	64/117 (54%)	Gap:	8/117 (6%)	Coverage:	41%
SefJ	Adhesin (*P. columbae*)						WP_100158730.1	
197 AA residues	Length:	275	Range:	153–273	Eval:	4.00E‐05		
	Ident:	39/124 (31%)	Pos:	66/124 (53%)	Gap:	6/124 (4%)	Coverage:	61.0%
	Adhesin (*Proteus mirabilis*)						WP_088493555.1	
	Length:	275	Range:	153–273	Eval:	2.00E‐04		
	Ident:	39/123 (32%)	Pos:	65/123 (52%)	Gap:	4/123 (3%)	Coverage:	61%

*Note*: Hypothetical proteins and redundant proteins were omitted.

Abbreviation: CFU, colony forming unit.

**Figure A5:**
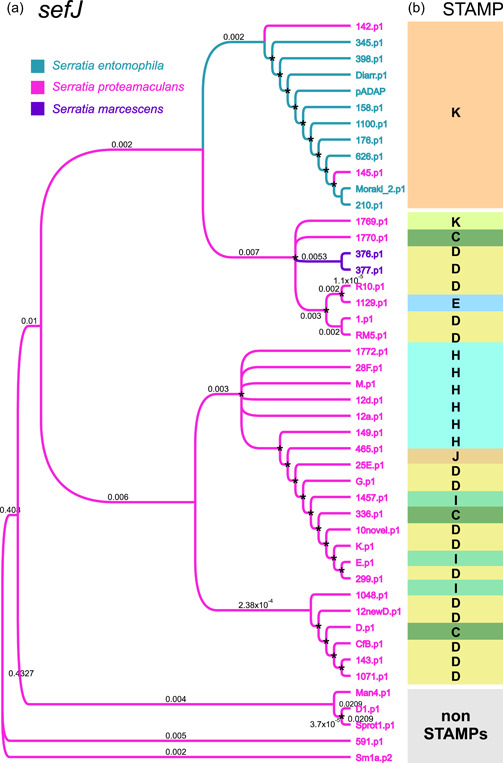
Comparison between *sefJ* divergence and the *Serratia* transmissible adaptive mega‐plasmids (STAMP)‐type encoding it. (a) Phylogenetic cladogram of nucleotide alignment of *sefJ*. Values on branches with 0 substitutions per site were hidden for clarity. (b) STAMP and non‐STAMP types as previously determined based on variation in the accessory regions of the plasmids (Sitter et al., 2021). There is a clear correlation between several clusters of *sefJ* sequences and the Tc encoding STAMP plasmid and bacterial species. *Denotes nodes with <75 bootstrap support, mostly due to sequences being identical.

**Figure A6:**
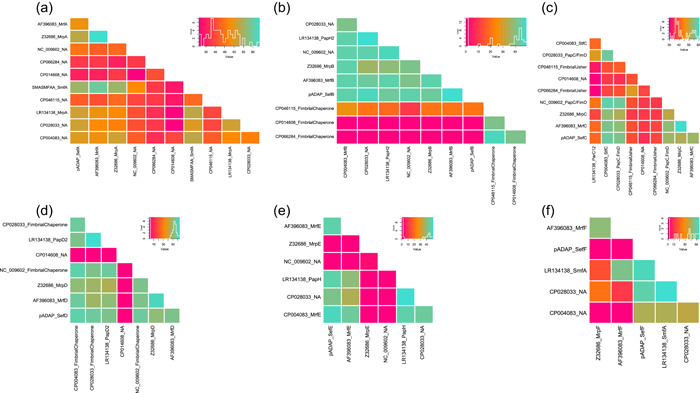
Distance matrices showing the amino acid identity in percentage between the Sef elements and their corresponding orthologs based on the homologous fimbrial operons identified in Figure 4. (a–f) These correspond to the SefA–F proteins. Colors per plot denote different sequence similarity ranges within a protein and are not directly comparable to different proteins.

Of note, SefD and SefJ appear to be the least conserved (Figure 2; Table 1) which may reflect their function. As SefJ is the predicted fimbrial tip it may differ in target specificity, and SefD is the chaperone that aids assembly of Sef components including SefJ through the cell wall. These differences may reflect species‐specific lineages. A phylogenetic comparison of the *sefJ* genes shows a strong correlation to the plasmid and bacterial species that encodes and therefore may reflect recognition of self, for example, to facilitate conjugation of the Sef encoding plasmid, or occupy a species‐specific ecological niche (Figure A5).

Though we have yet to define the substrate to which SefJ binds, based on the predicted role of SefJ as an adhesin, it is plausible that Sef facilitates colonization in the *C. giveni* larvae gut while Tcs induce chronic amber disease, both helping the bacterium proliferate.

### 2.4 Expression and deletion of *sef* operon confirms fimbriae production

**Figure 5:**
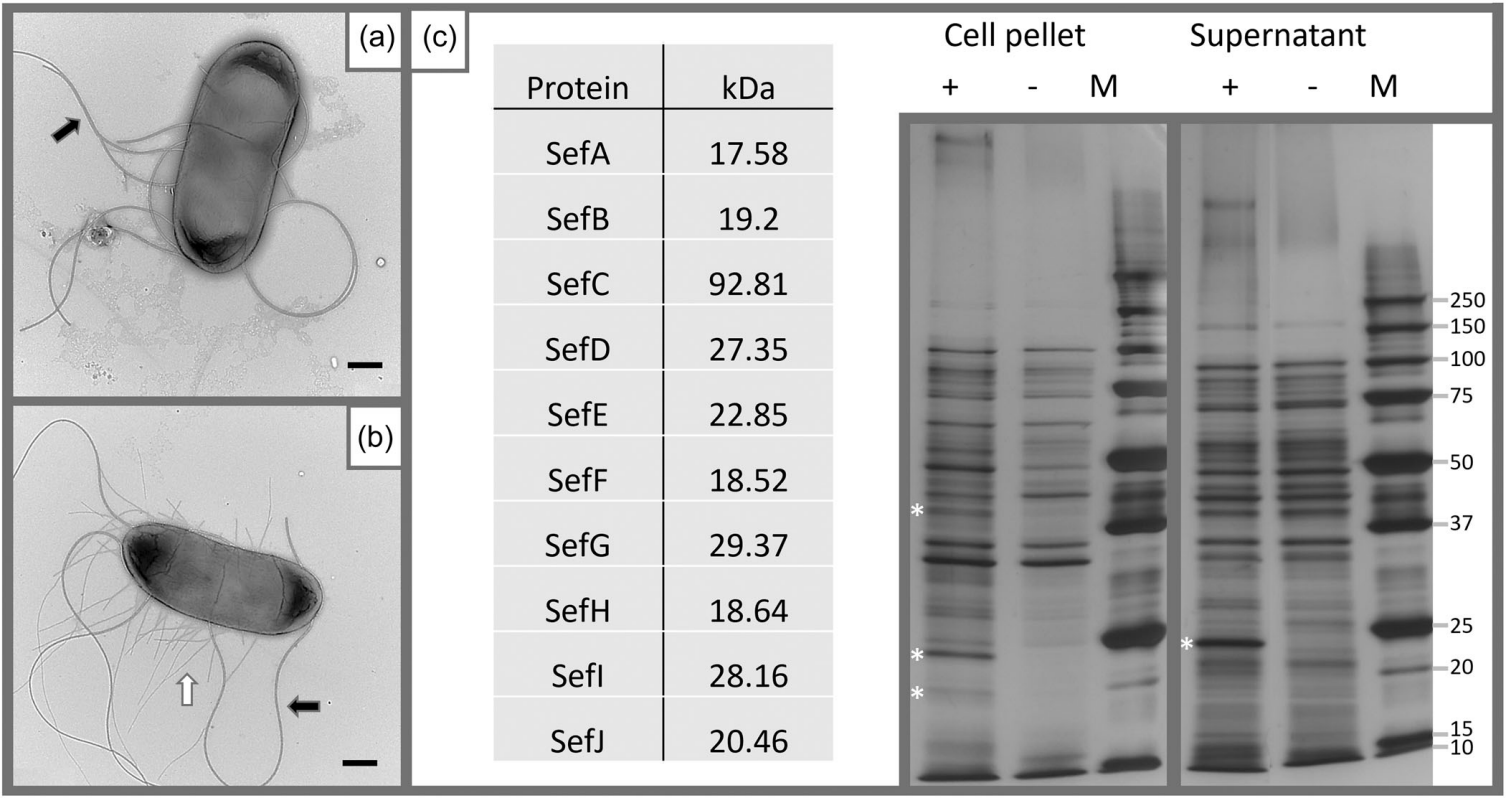
Expression of Sef in a *fim* null *Escherichia coli* strain. (a) Electron micrograph of the Δ*fimA*–*H E. coli* strain AAEC072A (pAY2‐4). (b) Electron micrograph of AAEC072 (pARA_SEF). White arrows denote Sef, and the black arrow denotes flagellum. (c) Predicted kDa of the listed Sef proteins; Sodium Dodecyl Sulfate‐Polyacrylamide gel electrophoresis (SDS‐PAGE) 10% of culture supernatant and cell pellet (1:100) at 24 h, Δ*fimA*–*H E. coli* strain AAEC072A (Blomfield et al., 1991) containing empty arabinose induction vector pAY2−4 (−), and the Sef expression vector pARA_SEF (+), compared to Bio‐Rad Precision Plus Protein Standards (M). Asterisk denotes the location of predicted fimbrial proteins, refer to table for predicted mass. Scale bars = 500 nm.

To investigate whether the *sef* operon produces fimbria the *sefA‐J* gene cluster was artificially expressed in the *E. coli fim* null derivative AAEC072A (Blomfield et al., 1991), using an empty pAY2‐4 (Shaw et al., 2003) vector and compared against an isolate carrying a Sef encoding pAY2‐4 mutant (pARA_Sef). Sodium Dodecyl Sulfate‐Polyacrylamide gel electrophoresis (SDS‐PAGE) showed the pARA_Sef bearing *E. coli* expressing several proteins of expected sizes to those of Sef (Figure 5c) and the formation of fimbria by *sef* expression was visually confirmed by TEM (Figure 5b). Analysis of the TEM images (Figure 5b) found the expressed fimbriae to average 756 nm in length (range 132–2732 nm) (Figure A8, Supporting Information S1: Table S8), with no fimbriae observed in the control, where only flagella were observed (Figure 5a). The data confirmed that the *sef* operon encodes fimbriae.

To determine if the *sef* cluster has a potential role in virulence, a pADAP *sefA*–*C* deletion variant A1MO2 (pADAP*ΔsefA*–*C*) was constructed and its ED_50_ (effective dose) compared to A1MO2 (pADAP) WT by challenging *C. giveni* larvae with these strains. No significant difference between the Sef‐producing WT A1MO2 and the *sefA‐C* deletion mutant was identified at day 12 post‐challenge for each strain based on the pADAP bearing A1MO2 WT control, with the amber disease typically observed 3–5 days postchallenge with the WT control (Table 2).

**Table 2:** Percent disease and mortality at Day 12 of *Costelytra giveni* larvae challenged with different amounts of CFUs of A1MO2 WT or the pADAPΔsefA–C mutant.

Strain	Blank	pADAP	pADAP	pADAP	pADAP	pADAP	pADAPΔ*sefA*–*C*	pADAPΔ*sefA*–*C*	pADAPΔ*sefA*–*C*	pADAPΔ*sefA*–*C*	pADAPΔ*sefA*–*C*
Spiked CFUs		10^7^	10^6^	10^5^	10^4^	10^3^	10^7^	10^6^	10^5^	10^4^	10^3^
Disease	0.0% ± 0.0%	91.7% ± 5.8%	66.7% ± 9.8%	50.0% ± 10.4%	4.2% ± 4.2%	4.2% ± 4.2%	87.5% ± 6.9%	75.0% ± 9.0%	45.8% ± 10.4%	4.2% ± 4.2%	0.0% ± 0.0%
Mortality	0.0% ± 0.0%	8.3% ± 5.8%	4.2% ± 4.2%	0.0% ± 0.0%	4.2% ± 4.2%	4.2 ± 4.2%	12.5% ± 6.9%	12.5% ± 6.9%	0.0% ± 0.0%	0.0% ± 0.0%	0.0% ± 0.0%

*Note*: Blank contains only miliq bidest H_2_O. Averages were calculated over two independent bioassays or 12 larvae each, 24 in total.

**Figure A7:**
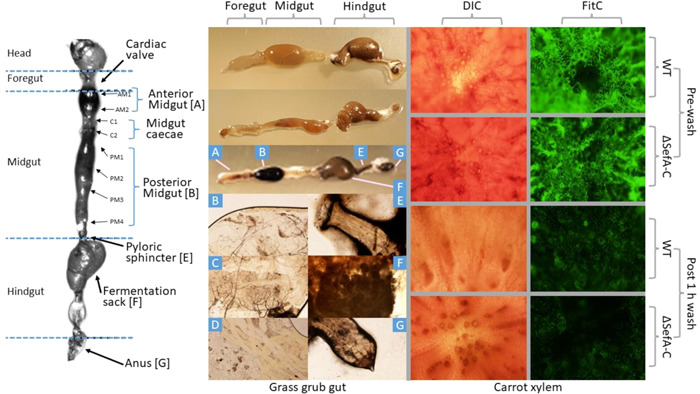
Lack of adhesion of Sef‐bearing bacteria in the gut of *Costelytra giveni*. Left panel, a schematic of the *C. giveni* larvae gut, divided into foregut, midgut, and hindgut. Microinjection of fluorescent A1MO2 with pUC30TGFPMut was performed, and on several days postingestion, the larval gut was extracted and studied, but in none of the cases adherence was observed and the bacterium was only observed in the gut juices. Additionally, an adherence experiment on carrots showed no difference in binding affinity between the A1MO2 (pUC30TGFPMut) or pADAP6se/A‐C bearing A1MO2 (pUC30TGFPMut).

**Figure A8:**
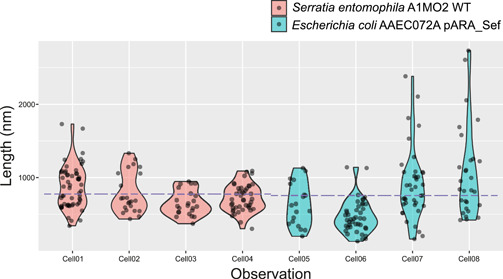
Fimbrial length distribution from four different cells (CELL01–4) for both the A1MO2 WT and the *Escherichia coli* Δ*fimA*–*H* AAEC072A strain (CELL05–08) transformed with pARA_SEF. The measured A1MO2 fimbrial lengths range from 302 to 1730 nm with an average length of 776 nm with a range. The measured *E. coli* strain AAEC072A (pARA_SEF) fimbrial lengths range from 132 to 2732 nm with an average of 756 nm. Measurements were made using ImageJ.

Attempts to define differences in colonization between WT and pADAPΔ*sefA*–*C* using chromosomally GFP‐tagged strains did not reveal any site of colonization in the *C. giveni* larvae gut (Figure A7), which is in line with previous studies (Hurst & Jackson, 2002). Based on the phylogenetic clustering of *sef* with plasmid and species, we could not rule out the possibility that Sef may facilitate the proliferation of Tc‐encoding plasmids within *Serratia* populations through bacterial cell‐to‐cell adhesion, thus increasing the chance for conjugation. However, there was also no observable difference in conjugation rates between WT pADAP and a pADAP*ΔsefA*–*C* bearing mutant, towards a pADAP devoid Tetracycline resistant A1MO2 derivative donor strain (5.6_Chrom_Tc). Additionally, using minimal medium and LB at either 15, 25, or 30°C no visual difference was found in biofilm formation of the pADAPΔ*sefA*–*C*, WT, and 5.6 as determined through crystal violet assay. The recently published *S. entomophila* 626 chromosome (RefSeq: NZ_CP074347.1) encodes five chromosomally encoded fimbria operons. The A1MO2 chromosome is assumed to be highly similar to 626, which may complicate adhesion experiments. Additionally, determining if there are triggers that transcribe the *sef* operon requires additional experiments, as TEM analysis only observed ~10% of WT A1MO2 cells exhibiting fimbria.

Although our completed assays revealed no direct correlation between *sef* and the bacterial pathogenic response, or maintenance of the Tc encoding plasmids within the population, the conserved co‐location of *sef* and *tcs* could have a symbiotic function in an as yet unidentified insect host or ecological niche. Further experiments need to be undertaken to narrow down the exact reason for this conserved *sef*‐*tc* island.

## 3 CONCLUSIONS

This study assessed the contribution of a Sef encoding operon to the pathogenic activity of Tc encoding *Serratia*. The translated products of s*efA‐J* share homology with elements from the *E*. *coli* type 1 fimbria Fim, mannose‐resistant Mrf fimbriae, Stf fimbria identified in *S. enterica* (Hurst et al., 2003), and adhesins found in *Proteus columbae*, *Proteus mirabilis*, and *Morganella morganii*. The divergence of SefJ putative adhesins and its correlation with Tc type and *Serratia* species suggests there is species‐specific selective pressure on both the Tc and Sef.

While the *Serratia* Tcs share a common ancestor with the *Y. frederiksenii* 49.p1 TcYF, the newly identified non‐STAMP plasmids that encode for Tcs share ancestry with several Yersiniaceae plasmids such as *Y. enterocolitica* LC20 plasmid1_80K, based on *repA a*nd backbone nucleotide sequence similarity. This suggests that the co‐location of the *sef* and *tc* clusters may have occurred at a distant time point. Extensive assessments of the NCBIs RefSeq database yielded no other example of Sef‐like fimbriae associated directly with a Tc. Further support for this is the absence of any *sef* colocated with other pathogenic islands such as Afp or other STAMP accessory determinants such as RUF. These findings also suggest that at one point in time, the *sef*‐*tc* island might have been horizontally transferable and faced its own unique evolutionary track.

The previously proposed model of STAMP evolution suggested that *sef* and its corresponding *tc* has been acquired in two separate instances by the STAMP backbone (Sitter et al., 2021), and was one of the first accessory regions acquired by the STAMP backbone. The data presented here corroborates that the *sef*‐*tc* island likely existed before becoming associated with the STAMP backbone.

The high correlation between Sef and Tc encoding regions and their high synteny preservation of the island suggests selective pressure favors preserving both elements. Our experimental designs have not yielded any hints at the possibility of a mutually beneficial function of Sef and Tcs. The leading hypothesis was that Sef somehow proliferates the plasmid through aiding conjugation, allowing more cells to produce Tcs; however, conjugation rate experiments have shown no such effect. Recent data have found that *Yersinia* Holin (lysis cassette), which is associated with secreting Tcs, is only expressed in a subset of the population (<10%) (Schoof et al., 2023). Through TEM, it was established that only ~10% of WT *S. entomophila* A1MO2 cells were observed to be fimbriated. Based on this, it is plausible that our inability to define Sef functionality relates to the potential expression of the *sef* operon only occurring in a subpopulation, making visualization of adhesion difficult to detect. Based on the role of fimbriae in other systems, Sef likely plays a yet‐to‐be‐determined role in the lifestyle of Tc‐encoding *Serratia*. The Sef may enable adhesion to a substrate in the soil or on a plant close to where *C. giveni* larvae habituate, optimizing the chance for the bacterium to be ingested by the larva. It is also possible that these pathogens are associated with additional insects that have not been studied yet. These avenues need to be investigated further to identify the reason behind this selective pressure to keep the Sef fimbria and the toxin complex operons together.

## 4 MATERIALS AND METHODS

### 4.1 Strains, vectors, and culture general conditions

Strains and plasmids used or constructed throughout the study are listed in Supporting Information S1: Tables S1 and S5. Primers and amplicons can be found in Supporting Information S1: Table S6. Culturing of isolates was undertaken in Lennox L Broth Base (LB‐broth, Invitrogen) liquid medium (Luria & Burrous, 1957). *Serratia* cultures were grown at 30°C and for *E. coli* at 37°C on a Ratek orbital incubator at 200 rpm. Antibiotics, substrates, and their concentrations used in the study are listed in the Supporting Information S1: Table S7. Unless stated otherwise, constructs for cloning were made using a two‐step fusion PCR approach as described by Atanassov et al. (2009) and Szewczyk et al. (2006). Amplicons and fusion products were ligated into pGEM®‐T Easy cloning vector (Promega Corp.) and transformed into Dh10ß competent cells (Durfee et al., 2008). Recombination vectors were transformed into ST18 supplemented with 5‐aminolevulinic acid. Mutants were PCR and sequence validated using validation primers listed in Supporting Information S1: Table S6. DNA manipulations and cloning were performed as described by Sambrook and Russell (2001). Plasmid DNA and PCR products were purified using the High Pure Plasmid Isolation Kit (Roche), and High Pure PCR Product Purification Kit (Roche) respectively, following the manufacturers' specifications.

### 4.2 Sef arabinose‐induction

For induction, both the AAEC072A containing an empty pAY2‐4 and AAEC072A containing a Sef encoding pAY2‐4 mutant, designated pARA_Sef, were grown in 40% LB broth to reduce the amount of the glucose inhibitor and supplemented with 200 µg/ml arabinose, shaken at 40 rpm at room temperature ~22°C for 24 h.

### 4.3 SDS‐PAGE assessment for the presence of Sef components

At the 6‐ and 24‐h time points, the cell pellet and culture supernatant of the arabinose‐induced AAEC072A cultures were assessed by SDS‐PAGE. SDS‐PAGE was undertaken following the method of (Laemmli (1970). SDS‐PAGE gels (10%) were run at 200 V for 50 min. SDS‐PAGE gels were stained using the “Short silver nitrate staining” protocol (Chevallet et al., 2006). Bio‐Rad Precision Plus Protein Standards marker was used.

### 4.4 TEM/Confocal microscopy

Electron Microscopy Sciences EMS200‐Cu Plastic‐coated 200‐mesh copper grids were coated with a 3 nm layer of carbon for better heat dispersal followed by glow discharge treatment. Three microliters of induced WT and pARA_Sef bearing AAEC072A cultures were added to the grid and left for 60 s then the residual liquid was removed using Whatman #1 filter paper. Samples were then negatively stained using 3 μL of 0.7% uranyl acetate for 45 s. Excess stain was again removed using the Whatman #1 filter paper and the grid was left to dry for at least 30 min. Grids were examined in a Morgagni 268D TEM, images were captured using an Olympus Megapixel III digital camera. Fimbrial lengths were measured using ImageJ v1.52d (Schneider et al., 2012)

### 4.5 Bioassays assessment

Pathogenicity was determined based on oral challenge bioassays (Glare et al., 1993), with varying culture concentrations tested on field‐collected *C. giveni* larvae. Blank contains only miliq bidest H_2_O, other samples contain 10 µL of an overnight culture. The dilutions represent estimated cells per milliliter of overnight culture based on overnight grown plates from the same dilutions combined with OD_600_ measurements taken using a SmartSpec™Plus (BioRad) UV spectrometer. The final assessment was based on the observed state of 12 larvae at Day 12 (Jackson & Saville, 2000). Bioassays were performed in duplicate (24 larvae in total per tested condition).

To assess the potential role of Sef in the grass grub gut, bacterial cultures were injected directly into the gut using a custom‐made in‐house micro pipettor before feeding, to eliminate the food as an adherence factor for the Sef/bacteria. Approximately three pulses of 0.1 mL of 1:10 diluted culture (~10^7^ colony forming unit [CFU]) were injected directly into the foregut of the grass grub larvae, using a blunted needle. Post injection, the larvae were placed back in clean trays and left overnight at 15°C to recover. The following day the gut was removed from each larvae and opened. Samples were examined using an Olympus BX53 microscope. The FitC channel was used to filter the emitted and excited light on the right wavelength for GFPmut3‐carrying samples. Emitted light for GFPmut3 mutants was produced with a CoolLED pE‐300. Both DIC and FitC Images were recorded with an Olympus DP74 fluorescent digital camera. Fluorescent images were taken at ×100 magnification at 5 ms exposure unless stated otherwise, DIC images were taken at ×100 magnification with exposure time being automatically determined by the software.

### 4.6 Conjugation experiments to assess Sef effect on conjugation

Both the Chloramphenicol resistant A1MO2 with pADAP_Cm reporter plasmid and the A1MO2 (pADAP*ΔsefA*–*C*) were separately plated with a chromosomally tagged plasmid minus 5.6 mutant (5.6_Chrom_Tc) on a LB‐agar medium plate for conjugation. The chromosomal tag in 5.6 conveys resistance towards Tetracycline. LB broth was seeded with *S. entomophila* strains and grown overnight for ~16 h. The following morning the OD 600 nm of the culture was measured in conjunction with plating out a dilution range of the cultures, enabling the determination of CFU per milliliter. Using the measured OD, an equal amount of CFU for recipient 5.6 strain and respective WT and pADAP*ΔsefA*–*C* mutant donor were plated on regular LB‐agar and incubated at 30°C for ~6 h. The lawn was resuspended in 1 mL of LB broth, and serially diluted, 10 μL of the 10^–6^ through 10^–9^ dilutions were plated on LB‐agar plates; selective for the transconjugants and the plates incubated overnight at 30°C. Two hundred prospective transconjugants were then patched to validate sensitivity to the donor chromosomal antibiotic marker. Conjugations were performed five independent times

### 4.7 Biofilm formation experiments

Three milliliters LB broth cultures of A1MO2, the A1MO2 (pADAP*ΔsefA*–*C*) derivative, and the isogenic plasmid cured strain 5.6 were grown overnight without antibiotics at 30°C. One milliliter of the culture was then pelleted (6000 g, 5 min) and the cell pellet resuspended in 1 mL M9 media. Per treatment 5 µL of the M9 cell resuspension was pipetted into 12 independent wells (2 × 8 replicate well columns) of a 96‐well plate containing 195 µL aliquots of either M9+ glucose, M9+ casamino acids or LB broth. The plates were sealed with an Excel Scientific Inc, Aera Seal cat BS‐25 membrane and then placed at either 15, 25, or 30°C in a Ratek orbital shaker at 250 rpm for 24 h. The biofilm assay was performed in triplicate on different days. Biofilm formation was then visually assessed using the method of O'Tool (2011) and the isolates compared to the wildtype A1MO2 strain.

### 4.8 Bioinformatics

DNA of 76 isolates was processed as described by Sitter et al. (2021). Additionally, the previously sequenced pADAP reference genome (NC002523) (Hurst et al., 2011) was used as a reference. Non‐STAMPs encoding for Sef were identified in this study through mapping contigs to a local reference database of known STAMP features using BlastN (Camacho et al., 2009). This strategy yielded six non‐STAMP plasmid contigs encoding Sef and Tcs.

Annotation of plasmid contigs was performed using Prokka (Seemann, 2014). Scripts outlining the parameters and settings used for assembly and annotation at available in GitHub: https://github.com/IamIamI/pADAP_project/tree/master/Bash_scripts. Alignments of regions of interest were generated using ClustalW (Larkin et al., 2007). ML phylogenetic trees (×1000 bootstrapped) were constructed from aligned nucleotide sequences using IQ‐TREE (Chernomor et al., 2016). Prediction of gene function based on protein structure homology was generated using the Phyre2 (Kelley et al., 2015) and InteProScan (Jones et al., 2014) web services.

Distance matrix figures were generated using custom R scripts, written using R Studio 1.1.463 (RStudio: Integrated Development for R v1.2.1335 build1379, Vol. 1, 2015) running R 3.5.3 (R: A language and environment for statistical computing v3.6.1, Vol. 3, 2019), available in GitHub: https://github.com/IamIamI/pADAP_project/tree/master/Distance_Matrices and were manually adjusted with CorelDraw X8 (CorelDraw v19.0.0.0328 x86, Vol. X8, 2016) to stylize results, as well as label *x*‐and *y*‐axis to show species to which the plasmid belong.

## AUTHOR CONTRIBUTIONS

**Lesley Sitter**: Conceptualization (lead); data curation (lead); formal analysis (lead); investigation (lead); methodology (lead); resources (lead); software (lead); validation (lead); visualization (lead); writing—original draft (lead); writing—review and editing (lead). **Marion Schoof**: Formal analysis (supporting); investigation (supporting); methodology (supporting); writing—review and editing (equal). **Travis R. Glare**: Conceptualization (equal); funding acquisition (equal); project administration (equal), resources (equal); supervision (equal); writing—original draft (equal). **Murray P. Cox**: Funding acquisition (equal); supervision (supporting); writing—review and editing (equal). **Peter C. Fineran**: Funding acquisition (equal); supervision (supporting); writing—review and editing (equal). **Paul P. Gardner**: Funding acquisition (equal); supervision (supporting); writing—review and editing (equal). **Mark R. H. Hurst**: Conceptualization (equal); funding acquisition (equal); project administration (equal); resources (equal); supervision (equal); writing—original draft (equal).

## CONFLICT OF INTEREST STATEMENT

None declared.

## ETHICS STATEMENT

None required.

## Supporting information

Supporting information.

## Figures and Tables

**Figure A4:**
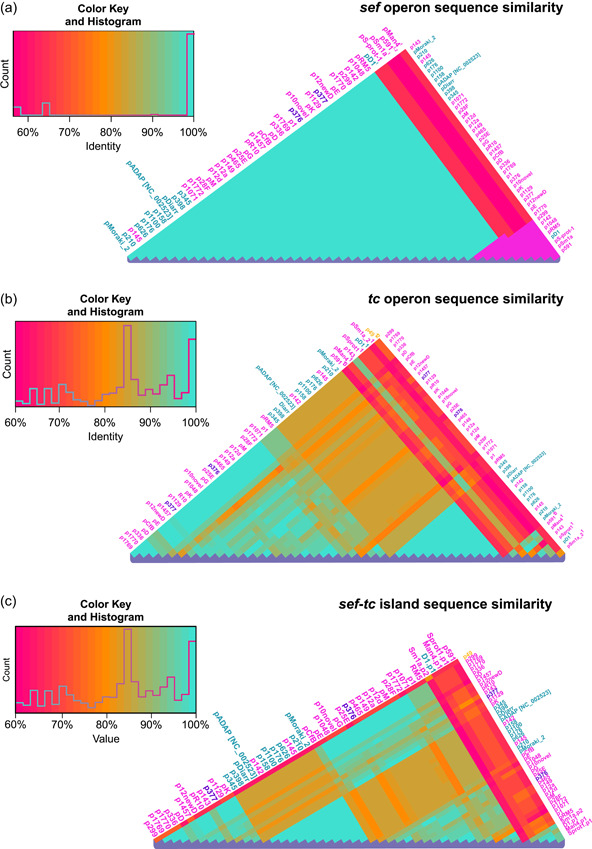
Distance matrix of all Tc/Sef encoding regions analyzed in this study.

## Data Availability

All processed sequencing data generated in this study have been submitted to the NCBI Reference Sequence database (RefSeq) under accession numbers MT039142–MT039228 (Supporting Information S1: Table S1).

## 1

Figure A4

**No functionality defined for Sef**

Potentially, Sef aids adherence to a host and assists colonization of the gut of the *C. giveni* larvae. Several attempts have been made to define a site of colonization of *S. entomophila* in *C. giveni* larvae (Hurst & Jackson, 2002), but have been unsuccessful. To determine whether we could observe any sites of colonization aided by Sef, A1MO2 bearing a GFP‐encoding vector (pUC30TGFPMut) in parallel with either pADAP or pADAP□se/A–C (pUC30TGFPMut) were independently assessed for adherence either within the grass grub gut or on cut carrot using fluorescent microscopy. Similar to previous studies, no site colonization was observed in any regions of the larval gut, as depicted in Figure A7. Instead, only free‐floating (no cell aggregates) fluorescent cells were observed, corroborating previous results reported by Hurst and Jackson (2002). No difference in fluorescence was observed between either A1MO2 carrying pADAP or pADAP□se/A–C on diced carrot (Figure A7). Another hypothesis is that sef may interact with other bacteria, through mediating cell–cell adherence, which in turn may facilitate pilus‐mediated conjugation. A comparative conjugation assay was performed using either the chloramphenicol‐resistant A1MO2 pADAP□sefA–C (seJ‐) or the kanamycin‐resistant A1MO2 pADAP _Afp2::KmR (ser) donors, and independently conjugating their plasmids to the spectinomycin resistant 5.6 plasmid‐free recipient strain. Selection of pADAP□sefA–C or pADAP _Afp2::KmR transconjugants found no difference in the number of transconjugants between the sef+ (pADAP _Afp2::KmR) or sef‐ (pADAPruefA–C) strains.
